# Supplementary material for: Leveraging prior knowledge to infer gene regulatory networks from single-cell RNA-sequencing data
Source: Mol Syst Biol. 2025 Feb 12;21(3):214–30. doi: 10.1038/s44320-025-00088-3 (PMC11876610; doi:10.1038/s44320-025-00088-3)
Supplement: Supplementary file 1 — Appendix [file 44320_2025_88_MOESM1_ESM.pdf]

Appendix for 'Leveraging prior knowledge to infer Gene Regulatory Networks from single-cell RNA-sequencing data'

Below, we describe in further detail recent graph-prior-based algorithms, which cover the three "operation modes" we discuss in this review: GENELink (constructive), CellOracle (destructive), Inferelator 3.0 (constructive) and scGLUE (hybrid).

Contents

Appendix Text S1: GENELink 3

Appendix Text S2: scGLUE 4

Appendix Text S3: CellOracle 5

Appendix Text S4: Inferelator 3.0 5

Appendix Text S1: GENELink

GENELink is a prior-informed GRN inference method relying on a graph attention mechanism to infer GRNs through a link prediction framework. This model allows predicting TF-TG relationships by exploiting a low dimensional embedded representation of genes retrieved from graph-based prior knowledge and gene expression data. Being $X \in R^{N \times M}$ a scRNA-seq count matrix and $A \in R^{N \times N}$ an adjacency matrix from a source of prior knowledge on genes interactions, with N number of genes and M number of observed cells, such learnable embedding is identified through a mapping function $E = f_{\phi}(X, A)$, where ϕ represent the set of learnable parameters of the model. GENELink is composed of two graph attention layers, producing an internal gene vectorial representation from the original space of observations (cells) that allows for capturing higher-order neighbours' information. Being $\overrightarrow{g_i}$ the vector of observations for gene i and N_i his neighbours in the prior graph (self-loops can also be included), the first graph attention layer transforms it as

$$\overrightarrow{g_i^1} = ||_{k=1}^{K} \sigma \left(\sum_{j \in N_i} \alpha_{ij}^k W_k^T \overrightarrow{g_j} \right) \tag{1}$$

with σ as an *elu* (exponential linear unit), $||_{k=1}^{K}$ a concatenation operator over K independent attentions as multi-head attention mechanisms, W_k^T learnable weights matrices and α_{ij}^k attention parameters, obtained as

$$\begin{gathered} att_{i,j}^k = \mathbf{a^k} \left(W_k^T \overrightarrow{g_i}, W_k^T \overrightarrow{g_j} \right) \\ \alpha_{ij}^k = softmax_{j \leftarrow N_i} \left(att_{i,j}^k \right) = \frac{exp\left(LeakyReLU\left(att_{i,j}^k\right)\right)}{\sum_{n \in N_i} exp\left(LeakyReLU\left(att_{i,n}^k\right)\right)} \end{gathered} \tag{2}$$

where $\mathbf{a}$ is the attention operation, while the softmax is applied over all neighbours for the i-th gene. Similarly, the second graph attention layer transforms the first hidden layer by averaging over multi-head attention:

$$\overrightarrow{g_i^2} = \sigma \left(\frac{1}{K} \sum_{k}^{K} \sum_{j \in N_i} \alpha_{ij}^k W_k^T \overrightarrow{g_j^1} \right) \tag{3}$$

From this latent representation, a further encoding is added to force the embedding to lower dimensions. Pairwise genes latent representations $\overrightarrow{g_i^2}$ are inputs of two MLPs channels composed of 2 layers:

$$\begin{gathered} \overrightarrow{e_i} = LeakyReLU \left(W_2^T LeakyReLU \left(W_1^T \overrightarrow{g_i^2} + b_1 \right) + b_2 \right) \\ \overrightarrow{e_j} = LeakyReLU \left(W_4^T LeakyReLU \left(W_3^T \overrightarrow{g_j^2} + b_3 \right) + b_4 \right) \end{gathered} \tag{4}$$

where $W_1^T - W_4^T$ are transposed linear weights and $b_1 - b_4$ biases of the MLPs layers.

Edges' labels are then obtained from the dot product of final nodes (genes) representation, and the objective loss is pointwise binary cross entropy over original edges labels y_i for TF-TG pairs:

$$score = \overrightarrow{e_i} \cdot \overrightarrow{e_j}$$

$$BCE = -\sum_i^K y_i log\left(GENELink_\Theta(x)\right) + (1 - y_i)\, log\left(1 - GENELink_\Theta(x)\right) \tag{5}$$

Finally, for edges causal inference, the embedding of gene pairs is concatenated and transformed by an external dense layer of in two or three probability nodes

$$\begin{gathered} \overrightarrow{h_{ij}} = W_e^T \left(\overrightarrow{e_i} || \overrightarrow{e_j}\right) + b_e \\ p^k = \frac{h_{ij}^k}{\sum_d h_{ij}^d} \end{gathered} \tag{6}$$

where p_k, for the case of d=3, represent respectively the probability of interaction $i \to j$, $j \to i$ and no interactions.

Appendix Text S2: scGLUE

scGLUE (graph-linked unified embedding) is a computational framework that is capable of combining different omics layers in order to model regulatory interactions. The structural setup is based on autoencoders. Assume $k = 1, ..., K$ different omics layers, each consisting of a distinct feature set $\mathcal{V}_k$ and data space $\mathcal{X}_k \subseteq R^{|\mathcal{X}_k|}$. The cells are denoted by $x_k^n \in X_k,\ n = 1, ..., N_k$ and the corresponding observations are $x_{k,i}^n, i \in \mathcal{V}_k$ for feature i. Using a prior distribution $p(\mathbf{u})$ a low-dimensional latent variable is introduced,

$$p(\mathbf{x}_k, \theta_k) = \int p(\mathbf{x}_k | \mathbf{u}, \theta_k) p(\mathbf{u}) d\mathbf{u} \tag{7}$$

Here θ_k are the parameters that should be learned. The latent variable $\mathbf{u}$ is shared across different omics layers.

The prior knowledge is induced through a knowledge graph $G = (V, E)$ which contains regulatory interaction at different omics layers, i.e. $V = \bigcup_{k=1}^K \mathcal{V}_k$ and $E \subseteq \{(i, j) | i, j \in V\}$.

The variational posterior in the autoencoder is given by a factorization

$$q(\mathbf{u}, \mathbf{V} | \mathbf{x}_k, G; \phi_k, \phi_G) = q(\mathbf{u} | \mathbf{x}_k; \phi_k) q(\mathbf{V} | G; \phi_G) \tag{8}$$

The graph encoder $q(\mathbf{V}|G; \phi_G)$ is modelled by a diagonal-covariance normal distributions whose parameters ϕ_G are inferred by a graph convolutional network. Similarly, the data encoder $q(\mathbf{u}|\mathbf{x}_k; \phi_k)$ is modelled by a diagonal-covariance normal distribution as well and the parameters ϕ_k are determined by multi-layer perceptrons.

The model likelihood including the prior knowledge G is

$$p(\mathbf{x}_k, G; \theta_k, \theta_G) = \int p(\mathbf{x}_k|\mathbf{u}, \theta_k) q(G|\mathbf{V}; \phi_G) p(\mathbf{u}) p(\mathbf{V}) d\mathbf{u} d\mathbf{V} \tag{9}$$

where θ_k and θ_G are the learnable parameters in the decoder that govern the learnable generative distributions for the omics data. The graph decoder in the model likelihood is

$$p(G|\mathbf{V}, \theta_G) = E_{i,j\sim p(i,j;w_{i,j})}[\sigma(s_{i,j}\mathbf{v}_i^T\mathbf{v}_j) E_{j'\sim p_{ns}(j'|i)}(1 - \sigma(\mathbf{v}_i^T\mathbf{v}_{j'}))] \tag{10}$$

where $\mathbf{V} \in R^{m\times|V|}$ is a matrix that combines all omics features and the edges (i, j) are sampled with probabilities proportional to the edge weights. The prior distributions for u and V are standard normal distributions. The data decoder is dependent on the type of omics data, but for count-based scRNA-seq and scATAC-seq data, a negative binomial distribution is inferred.

To ensure the proper alignment of different omics layers, the adversarial alignment strategy is applied, and the overall minimization is performed with respect to the multiclass classification cross-entropy.

Appendix Text S3: CellOracle

CellOracle is based on a regularized linear regression model. The expression of a target gene y is predicted by a linear combination of the gene expression of regulatory candidate genes x_i

$$y = \sum_i \beta_i x_i + \alpha \tag{11}$$

Here, the model parameters are β_i and α. The list of potential regulatory genes x_i is extracted from the prior knowledge network by taking all nodes that feature connections to the target gene of interest. The gene expression matrix of scRNA-seq data is divided into several clusters in advance in order to ensure that a single data unit represents a linear relationship. For the inference of the parameters, two approaches are suggested, namely Bayesian Ridge Regression or the Bagging Ridge Regression. Regularization is applied in order to prevent overfitting and identify informative variables. In both cases, a posterior distribution of coefficient value β is computed, which allows testing for the significance of the linear fit. The coefficients β_i are interpreted as edge strengths between a TF and its TG, where a higher value indicates higher dependence. The final selection of edges is based on thresholds for the p-values and absolute values of the β_i which can be defined according to the data type and quality as well as the goal of the analysis.

Appendix Text S4: Inferelator 3.0

The third version of the Inferelator 3.0 scheme is based on regression. By assuming that the connections contained in the prior knowledge are included

in the connectivity matrix P, the observed expression of gene j at sample i is modelled by

$$X_{i,j} = \sum_k A_{i,k} P_{k,j} \tag{12}$$

A stores the so-called *transcription factor activity* and is approximated by minimizing the following least squares problem

$$||\hat{\mathbf{A}}\mathbf{P} - \mathbf{X}||_2^2 \tag{13}$$

where $\hat{A}_{i,k}$ is the estimated latent transcription factor activity for sample i and transcription factor k. This matrix is then used to infer the following linear model,

$$X_{i,j} = \sum_k \hat{A}_{i,k} \beta_k \tag{14}$$

where β_k are the model parameters. This inference step remains generic and therefore can be carried out using different regression methods and can be adapted to feature regularization and bootstrapping to reduce the effect of outliers and sampling error. Gibbs et al. suggest the use of Bayesian Best Subset Regression and StARS-LASSO for this purpose.
